# Effects of Different Drying Techniques on the Post‐Harvest Quality of Fermented Cocoa Beans in Ghana

**DOI:** 10.1002/fsn3.70715

**Published:** 2025-08-27

**Authors:** Lukeman Haruna, Ernest E. Abano, Ernest Teye, Mary Lukeman, Isaac Tukwarlba, Wilson Yeboah, David Nukafo, Kesse J. Agyei

**Affiliations:** ^1^ Department of Agricultural Engineering University of Cape Coast Cape Coast Ghana; ^2^ Ghana Cocoa Board (COCOBOD) Quality Control Company Limited Sefwi Wiawso Ghana; ^3^ Food and Drugs Audit Food and Drugs Authority (FDA) of Ghana Accra Ghana; ^4^ Department of Plantation Cocoa Research Institute of Ghana (CRIG) New Tafo Ghana

**Keywords:** cocoa beans, convective oven drying, drying techniques, modified convective oven drying, open sun drying, volatile compounds

## Abstract

Drying plays a pivotal role in post‐harvest processing, influencing the quality, storability, and commercial value of cocoa beans. This study compared the impact of three drying techniques—open sun drying (OSD), convective oven drying (COD), and a modified convective oven drying method (MCOD), which emulates sun drying by controlling temperature and humidity—on the quality characteristics of fermented cocoa beans from Ghana. Key attributes assessed included pH, fermentation index (FI), fermentation quality (FQ), fat and polyphenol content, levels of free fatty acids (FFA), sugars, organic and amino acids, and the profile of volatile compounds. Beans dried using MCOD achieved the highest FI (1.16) and a higher FQ of 96.23%, closely mirroring results from OSD (FI = 1.14, FQ = 98.23%). COD produced the lowest values (FI = 0.90, FQ = 89.23%). Fat content was preserved in OSD and MCOD (52.82% and 52.03%, respectively), whereas COD resulted in a lower fat yield (40.29%). Total polyphenol content (TPC) peaked in COD samples (40.55 mg GAE/g), while MCOD had the lowest FFA content (0.77%). OSD and MCOD retained higher levels of sugars and free amino acids and showed richer volatile profiles. Principal component analysis revealed similar chemical profiles between OSD and MCOD, particularly in sugars, amino acids, and volatiles, while COD samples were more associated with organic acids. These findings suggest MCOD is a practical and scalable substitute for traditional sun drying, especially under unfavorable weather conditions. Further studies should examine its potential in retaining bioactives, minimizing mycotoxins, and preserving sensory attributes.

## Graphical abstract

Effects of cocoa drying techniques on bean quality: This study compares open sun drying (OSD), convective oven drying (COD), and a modified convective oven drying (MCOD) method on the quality of fermented cocoa beans. MCOD closely mimics OSD and preserves key chemical and sensory attributes—such as a high fermentation index, fat content, and volatile profiles—while COD results in higher levels of undesirable organic acids and a reduction in flavor precursors. The findings support MCOD as a viable and scalable alternative for improving cocoa quality in weather‐limited regions.

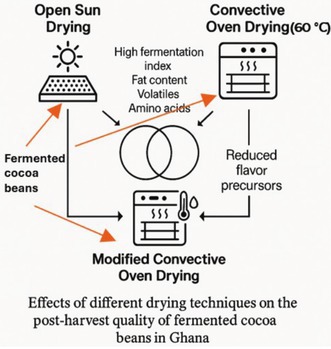

## 1 Introduction

Cocoa ( *Theobroma cacao* L.) stands as one of Ghana's most vital agricultural exports, playing a central role in the national economy and supporting the livelihoods of countless smallholder farmers (Kolavalli and Vignari 2011; Anele 2023). Beyond its economic contribution, cocoa serves as a core ingredient in chocolate production and has diverse applications in the food, pharmaceutical, and cosmetic industries (Ardhana and Fleet 2003; Abbadi et al. 2019). Maintaining high‐quality standards for Ghanaian cocoa is therefore essential for ensuring competitiveness in the international market (Thompson et al. 2007; Cudjoe et al. 2016).

Post‐harvest processing, particularly fermentation and drying, is critical to the chemical and sensory development of cocoa beans (Guda and Gadhe 2017; Haruna et al. 2024b). During fermentation, complex biochemical changes occur that are essential for the formation of flavor precursors and pigmentation (Owusu et al. 2011; Arsène et al. 2016; Afoakwa 2014; Guehi, Dadie, et al. 2010). Drying, on the other hand, reduces bean moisture to safe storage levels while further influencing flavor development and shelf stability (Misnawi et al. 2003; Schwan and Pereira 2014; Adeigbe et al. 2021). Additionally, effective drying helps limit microbial spoilage and modulates the distribution and retention of volatile aroma compounds (Jespersen et al. 2005; Erazo Solorzano et al. 2023).

Various drying approaches are utilized to achieve desired moisture levels in cocoa, including natural or sun drying, solar‐assisted systems, and mechanical methods (Banboye et al. 2020). Due to its low cost and simplicity, sun drying remains the most widely adopted method among smallholder farmers (Sahdev et al. 2016; Adams et al. 2024). This method involves spreading fermented beans on raised platforms, mats, or concrete slabs for exposure to direct sunlight (Ewe 2015; Djikeng et al. 2018). The gradual drying process aids in moisture migration and acid volatilization while enhancing the internal distribution of flavor compounds formed during fermentation (Erazo Solorzano et al. 2023).

Despite its advantages, sun drying has notable limitations, such as its dependence on weather, vulnerability to contamination, and slow drying rates (Hii et al. 2009; Tardzenyuy et al. 2020). It also exposes beans to pilfering, discouraging proper drying and turning by farmers, often leading to quality issues like high bean acidity and inadequate flavor development (Afoakwa and Paterson 2010; Niikoi et al. 2022). Various studies have investigated alternative drying technologies for cocoa, including solar‐assisted dryers (e.g., tunnel and cabinet dryers), microwave‐assisted drying, and hybrid systems designed to accelerate moisture removal while minimizing quality loss (Abhay et al. 2017; Mujaffar et al. 2017; Banboye et al. 2020). Although these methods offer advantages in drying efficiency, they may introduce abrupt thermal gradients or oxidative stress that can affect flavor and chemical stability (Guehi, Zahouli, et al. 2010; Abano et al. 2011; Afoakwa 2014). In this study, a modified convective oven drying (MCOD) system was adopted to closely simulate the gradual and humidity‐influenced nature of sun drying. Unlike conventional convective systems, the MCOD was programmed using real‐time environmental data collected during sun drying, allowing for dynamic control of temperature and relative humidity. This approach ensures higher reproducibility, better hygiene, and potential scalability for laboratory and pilot‐scale applications, particularly in regions with limited or unreliable sunlight.

## 2 Materials and Methods

### 2.1 Materials

Hybrid 7 cocoa pods were harvested manually from experimental fields at the Cocoa Research Institute of Ghana (CRIG), located in New Tafo, Eastern Region (Site K8: Latitude 6.231906, Longitude −0.350051; Site G15: Latitude 6.230603, Longitude −0.349467). This hybrid was selected for its availability, wide cultivation, and reliable fermentation characteristics in Ghana.

#### 2.1.1 Pod Breaking, Pre‐Drying, and Fermentation

After harvest, the pods were manually broken to extract the beans, which were spread on perforated plantain leaves laid over bamboo platforms (dimensions: 2 m height, 1 m width, 3 m length) to allow mucilage drainage. Beans underwent initial sun exposure for six hours under ambient conditions (28.1°C ± 5.9°C; relative humidity: 81.8% ± 17.0%), with hourly stirring to ensure uniform dehydration. Post pre‐drying, 60 kg of beans were piled (~90 cm high) on moderately perforated black plastic sheets, which were sealed by folding and knotting the edges to form a compact heap (approximate size: 112 × 115 × 31 cm). Fermentation occurred spontaneously for six days with manual turning every 48 h and was replicated in triplicate.

#### 2.1.2 Drying Experiments

A representative 1.5 kg sample of freshly fermented beans was obtained through thorough mixing and quartering, then divided into three equal portions (≈500 g each) for subsequent drying using three techniques.

##### 2.1.2.1 Open Sun Drying (OSD)

Following slightly modified protocols from Hii et al. (2009) and Olabinjo et al. (2017), beans were spread in a single layer (1.03 cm thick) in a drying box lined with bamboo mats elevated 2 m above the ground. Drying was carried out for 8 h daily, with turning every 2 h to enhance uniformity. Temperature and humidity were recorded during each turning using a data logger (Elitech RC‐51, Elitech Technology Inc., California, USA). After each day's drying, samples were tempered overnight to redistribute moisture. Drying ended once the moisture content reached 7.5% (wet basis).

##### 2.1.2.2 Convective Oven Drying (COD *)*

Beans were spread in a single layer (1.03 cm thick) on meshed trays and placed in a conventional convective oven (Faithfull Model 101‐3AB, FCD‐300 series, China), preheated to 60°C following the method of Nwakuba et al. (2017). The oven operated on a forced convection mechanism, where a side‐wall‐mounted electric heater generated heat, and a fan circulated hot air uniformly across the drying chamber. Heated air passed over the cocoa samples, facilitating moisture removal through evaporation. The exhaust air escaped through a 4 cm diameter vent at the back of the oven, maintaining air turnover. Air velocity was maintained at 0.01 m/s. Beans were turned every 2 h over 8‐h drying cycles and tempered overnight. Drying continued until the beans reached 7.5% final moisture content (wet basis).

##### 2.1.2.3 MCOD

**FIGURE 1:**
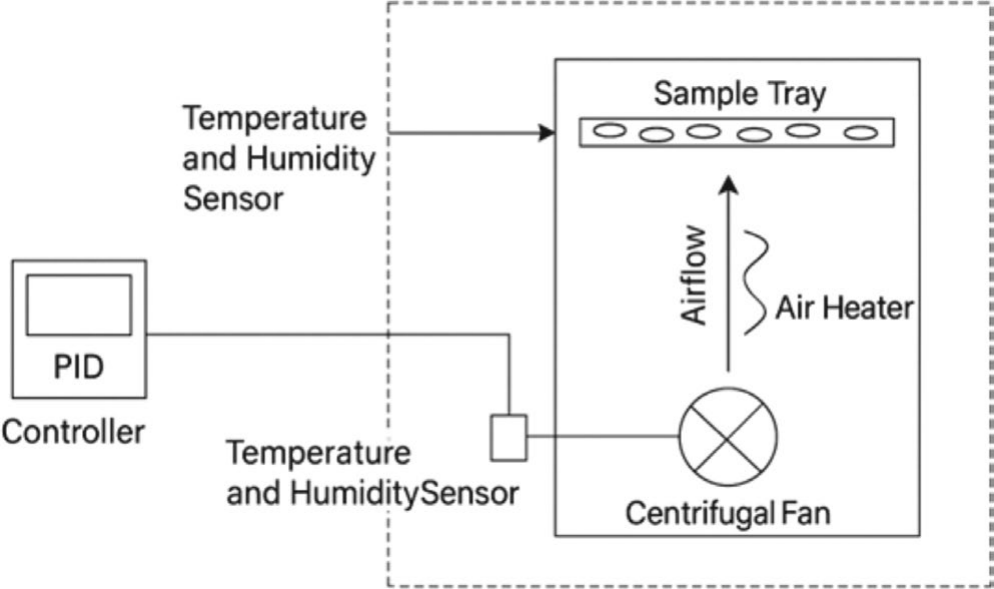
Schematic diagram of the MCOD system.

For MCOD, fermented beans were spread in a single layer (1.03 cm thick) inside a precision vacuum oven (Faithfull Model DZ‐BIV, China), equipped with a PID (Proportional–Integral–Derivative) control system. To closely mimic OSD behavior, the beans were placed into the oven under ambient conditions (temperature: 27.3°C ± 4.4°C; relative humidity: 84.2% ± 13.7%) before the drying program was initiated. This allowed the samples to experience the full diurnal progression of temperature and RH as recorded during natural sun drying. The heating system consisted of wall‐integrated resistive elements, while a low‐speed internal fan gently circulated air across the sample tray (Figure 1). The oven was programmed using real‐time environmental data collected during OSD, enabling dynamic simulation of natural temperature and RH fluctuations. Moist air was evacuated through a calibrated vent at the rear of the chamber to prevent humidity buildup. Drying was conducted for 8 h daily, with 2‐hourly turning and overnight tempering, and was terminated at 7.5% final moisture content (wet basis).

#### 2.1.3 Experimental Design

A completely randomized design (CRD) was adopted, with drying method (OSD, COD, and MCOD) as the main factor. Fermented but undried beans were included as control samples. Quality assessments included measurements of pH, fermentation index (FI), cut‐test scores, fat content, free fatty acid (FFA) levels, total polyphenol content, reducing sugars, free amino acids, organic acids, and volatile compound profiles.

### 2.2 Methods

#### 2.2.1 pH and FI Analysis

pH was measured following a modified version of the method described by Afoakwa, Kongor, et al. (2013). Ten grams of ground cocoa nibs were homogenized with 90 mL of distilled water and stirred for 30 min. The mixture was then filtered, and pH readings were taken using a calibrated digital pH meter (Model S213, Mettler Toledo, Geneva, Switzerland). Calibration was done using standard buffer solutions at pH 3.50, 7.00, and 9.50.

FI was determined spectrophotometrically using the absorbance ratio at 460 nm and 530 nm, according to García‐Alamilla et al. (2007). A 2.5 g sample of ground nibs was extracted with 50 mL of 0.1% HCl in methanol, shaken for 24 h, and filtered. Absorbance was measured using a UV/Vis spectrophotometer (Beckman Coulter DU 730 Life Science, MA, USA).

#### 2.2.2 Cut Test and Visual Quality Assessment

A total of 300 dried beans per treatment were longitudinally split and visually examined for internal coloration—classified as brown (fully fermented), purple (partially fermented), or slaty (unfermented). Defective beans, including moldy, germinated, and insect‐damaged specimens, were also recorded. The scoring approach followed Haruna et al. (2024a).

#### 2.2.3 Determination of Total Fat Content

Fat content was determined by Soxhlet extraction using AOAC Method 963.15 (2019), with petroleum ether (boiling point: 40°C–60°C) as the solvent. Five grams of ground cocoa were subjected to 8 h of continuous extraction using a Gerhardt Soxtherm system (Germany). Post‐extraction, the solvent was evaporated, and the fat residue was weighed to calculate fat content.

#### 2.2.4 FFA Analysis

FFA levels were assessed using the AOAC 940.28 (2019) titration method. A 5 g lipid sample was dissolved in 50 mL of neutralized ethanol and titrated with 0.1 N NaOH, using phenolphthalein as an indicator. FFA content was expressed as a percentage oleic acid equivalent.

#### 2.2.5 Determination of Total Polyphenol Content (TPC)

TPC was measured using the Folin–Ciocalteu colorimetric method (Albak and Tekin 2016), with adjustments. After fat removal by Soxhlet extraction, 250 mg of defatted powder was extracted three times with 40 mL of 80% aqueous acetone (acetone:water:acetic acid, 80:18:2, v/v/v). Each extract was sonicated in an ice bath for 30 min, then centrifuged at 1000 × g for 15 min. The combined extract was adjusted to 50 mL. For analysis, 0.5 mL of extract was reacted with 2.5 mL of 0.2 N Folin–Ciocalteu reagent and incubated with 7.5 mL of sodium carbonate solution. After 2 h in the dark, absorbance was read at 765 nm using a UV–Vis spectrophotometer (Beckman Coulter DU 730 Life Science, MA, USA). A standard curve was developed using a 50 mg stock solution of catechin (Merck, Darmstadt, Germany), with serial dilutions to obtain standard concentrations of 20, 40, 60, 80, and 100 μg catechin/mL.

#### 2.2.6 Quantification of Sugars and Organic Acids

Reducing sugars and organic acids were analyzed using HPLC, following Sari et al. (2023) with minor changes. Two grams of defatted cocoa powder were mixed with 6 mL of pre‐warmed ultrapure water, homogenized for 5 min, and centrifuged (13,000 rpm, 4°C, 10 min). The supernatant was treated with acetonitrile (1:4 dilution) to precipitate proteins, re‐centrifuged, and filtered (0.45 μm). Analysis was done using a Shimadzu LC‐20AB system with an Aminex HPX‐87H column (Bio‐Rad), RID‐10A detector, and 5 mM H₂SO₄ as the mobile phase (flow rate: 0.6 mL/min; column temp: 80°C). Calibration was performed using standard solutions of 20–100 μM, and the results were expressed as mg/g fat‐free dried sample.

#### 2.2.7 Determination of Free Amino Acids

FAAs were extracted as per Kirchhoff et al. (1989), with modifications. A 0.1 g sample was mixed with 0.3 g PVPP and 25 mL of ultrapure water, pH‐adjusted to 2.5 using 50% trifluoroacetic acid. The mixture was stirred at 4°C for 1 h, cooled in an ice bath, and centrifuged (5000 rpm, 10 min, 4°C). The supernatant was filtered (0.45 μm), lyophilized, then reconstituted in borate buffer. Amino acids were separated via HPLC using a LiChroCART 250‐4 column (Lichrospher 100 RP‐18) and identified using a fluorescence detector (excitation: 334 nm; emission: 425 nm). Quantification was based on peak area comparison with standard mixtures (1–10 pmol/μL). Results were expressed as mg/g fat‐free dried sample (ff‐sample). FAAs were classified into acidic (Asp, Glu, Asn, Gln), basic (His, Arg, Lys), hydrophobic (Ala, Phe, Tyr, Leu, Ile, Val), and other amino acids (Gly, Ser, Trp, Thr, Pro, Met).

#### 2.2.8 Volatile Compound Analysis

Volatile profiling was performed using headspace‐solid phase microextraction (HS‐SPME) coupled with GC‐MS, as described by Tran et al. (2015) and Rodriguez‐Campos et al. (2011). Five grams of ground cocoa were sealed in 20 mL headspace vials, equilibrated at 60°C for 30 min, and exposed to a DVB/CAR/PDMS fiber for 40 min. Desorption occurred in an Agilent 7890A GC injector at 250°C (splitless mode). VOCs were separated on an HP‐5MS column using helium (1.2 mL/min). The oven program ranged from 40°C (2 min hold) to 250°C in two ramps (6°C/min to 150°C, then 4°C/min to 250°C, 5 min hold). The detector was an Agilent 5975C MS in EI mode (70 eV, scan range: 50–550 m/z). Identification was based on mass spectra and retention indices using the NIST 17 database, with similarity thresholds ≥ 85%.

### 2.3 Statistical Analysis

Data were analyzed using Minitab 19.1 (MSS LLC, USA). Normality was confirmed via the Kolmogorov–Smirnov test, and variance homogeneity was evaluated using the modified Levene's test. One‐way ANOVA was used to assess treatment effects, followed by Tukey's post hoc test (or Games–Howell where assumptions were violated). Principal component analysis (PCA) was employed to explore the relationships between flavor‐related parameters. All analyses were done in triplicate, and results are expressed as mean ± standard deviation. Statistical significance was set at *p* < 0.05.

## 3 Results and Discussion

### 3.1 Effect of Drying Methods on pH of Cocoa Beans

The pH values of cocoa beans varied significantly (*p* < 0.05) across different drying methods (Table 1). Beans subjected to OSD had the highest average pH (5.49 ± 0.04), followed by those treated with MCOD (5.24 ± 0.11), and then COD (5.06 ± 0.04). The lowest pH was observed in the undried control (4.97 ± 0.21). These findings confirm previous reports that drying helps neutralize bean acidity, likely through volatilization of acidic compounds (Afoakwa 2014; Tardzenyuy et al. 2020; Niikoi et al. 2022).

**TABLE 1:** Physicochemical and biochemical properties of cocoa beans under different drying treatments.

Quality parameter	Control	Drying treatment		
		OSD	COD	MCOD
pH	4.97^c^ ± 0.21	5.49^a^ ± 0.04	5.06^b^ ± 0.04	5.24^a^ ± 0.11
Fermentation index (FI)	1.03^ab^ ± 0.02	1.14^a^ ± 0.02	0.90^b^ ± 0.04	1.16^a^ ± 0.06
Cut‐test score (%)				
Slaty	0.00	0.00	0.00	0.00
Purple	15.10^a^ ± 0.35	1.77^c^ ± 0.05	10.77^b^ ± 1.75	2.23^c^ ± 0.50
Brown	84.77^c^ ± 2.16	98.23^a^ ± 0.5	89.23^b^ ± 1.75	95.80^a^ ± 0.17
Moldy	0.00	0.00	0.00	2.00 ± 0.30
Germinated	0.00	0.00	0.00	0.00
Fermentation quality (FQ)	84.77^c^ ± 2.16	98.23^a^ ± 0.5	89.23^b^ ± 1.75	96.23^a^ ± 0.92
Fat content (%)	49.95^b^ ± 1.21	52.82^a^ ± 0.7	40.29^c^ ± 2.19	52.03^a^ ± 0.93
Free fatty acid (%)	0.71^c^ ± 0.03	0.77^b^ ± 0.02	1.06^a^ ± 0.03	0.77^b^ ± 0.09
Total polyphenol (mg GAE/g dry weight)	41.63^a^ ± 0.96	38.13^b^ ± 1.32	40.55^a^ ± 1.20	38.80^b^ ± 0.46
Sugars (mg/g fat‐free sample)				
Sucrose	3.61^a^ ± 0.12	3.35^b^ ± 0.15	3.40^ab^ ± 0.19	3.20^b^ ± 0.16
Glucose	9.17^a^ ± 0.23	8.70^b^ ± 0.26	7.85^c^ ± 0.20	8.41^b^ ± 0.24
Fructose	10.11^a^ ± 0.18	9.26^b^ ± 0.25	8.31^c^ ± 0.29	8.96^bc^ ± 0.23
Organic acid (mg/g)				
Acetic acid	5.82^a^ ± 0.24	4.53^b^ ± 0.15	3.94^c^ ± 0.16	4.27^b^ ± 0.18
Lactic acid	2.11^b^ ± 0.08	2.10^c^ ± 0.09	2.72^a^ ± 0.10	1.88^d^ ± 0.11
Citric acid	0.75 ^a^ ± 0.05	0.60^b^ ± 0.11	0.48^c^ ± 0.03	0.58^b^ ± 0.13

*Note:* Means in a row with the same superscript letter are not statistically significant (*p* ≤ 0.05; Tukey's Post Hoc Test).

Abbreviations: COD, convective oven drying; Control, Fermented undried cocoa; MCOD, modified convective oven drying; OSD, open sun drying.

The elevated pH in OSD samples likely results from their extended exposure to ambient airflow and mild drying conditions, which promote gradual acid diffusion and evaporation. Comparable results were observed by Afoakwa, Kongor, et al. (2013), who also noted higher pH in sun‐dried beans due to prolonged acid loss. Interestingly, no significant difference (*p* > 0.05) was found between OSD and MCOD, suggesting that MCOD effectively simulates the acid‐reducing conditions of sun drying. By contrast, COD beans exhibited significantly lower pH, which may be attributed to rapid moisture removal that traps fermentation by‐products, particularly organic acids like lactic and acetic acid, within the bean matrix (Camu et al. 2007; Hii et al. 2009; Rodriguez‐Campos et al. 2011).

### 3.2 Effect of Drying Methods on FI, Cut‐Test Score, and Fermentation Quality (FQ) Value of Cocoa Beans

The FI, a widely used metric for gauging fermentation completeness, showed significant variation (*p* < 0.05) across drying methods (Table 1). MCOD‐treated beans recorded the highest FI (1.16 ± 0.06), followed closely by OSD (1.14 ± 0.02), while COD produced the lowest value (0.90 ± 0.04). The control sample—fermented but undried—had an intermediate FI of 1.03 ± 0.02, confirming adequate fermentation before drying.

Typically, an FI value ≥ 1 signifies well‐fermented beans, whereas values below 1 suggest under‐fermentation (Afoakwa et al. 2008; Caligiani et al. 2014). Low FI values are linked to high anthocyanin levels, which strongly absorb at 530 nm. During proper fermentation, these pigments undergo hydrolysis and oxidation, decreasing absorbance at 530 nm and increasing at 460 nm, thus elevating the FI (Kongor et al. 2013; Kyi et al. 2005). The superior FI values in MCOD and OSD treatments indicate that these drying conditions may prolong enzyme activity—particularly glycosidases and polyphenol oxidase (PPO)—which further drive the breakdown of anthocyanins to anthocyanidins during drying (De Brito et al. 2000; Nazaruddin et al. 2006). In contrast, the sharp drop in FI observed in COD‐treated beans suggests that rapid drying may have suppressed these enzymatic reactions prematurely, possibly due to thermal inactivation of PPO and other oxidation‐related enzymes (Kyi et al. 2005; Hii et al. 2009).

The cut test further confirmed notable differences in FQ among the drying treatments (Table 1). Beans dried under OSD and MCOD showed the highest proportion of brown beans—98.23% ± 0.50% and 95.80% ± 0.17%, respectively—indicating well‐executed fermentation. COD‐treated samples recorded a lower brown bean occurrence (89.23% ± 1.75%), while the control had the lowest (84.77% ± 2.16%). These values align with internationally accepted grading standards, including ISO 2451 (2017) and FCC (2023), which classify cocoa as well‐fermented when the proportion of brown beans is high (> 60%) and defects remain within acceptable limits—specifically, not more than 4% moldy, 4% slaty, and 8% for other defects such as insect damage, germination, or decay (Zahouli et al. 2010; Guehi, Zahouli, et al. 2010).

As shown in Table 1, the proportion of purple beans—indicative of partial fermentation—was highest in COD (10.77% ± 1.75%) and lowest in OSD (1.77% ± 0.05%). This suggests that rapid drying under COD conditions may have prematurely suppressed enzymatic and microbial activity, thereby inhibiting the complete breakdown of anthocyanins into tannins, which give rise to the brown coloration. Instead, pigment oxidation may have halted at the quinone stage, producing the persistent purple hue. These observations are in line with findings by Santander et al. (2025), who reported that high drying temperatures can interrupt fermentation‐related pigment degradation, resulting in a higher proportion of incompletely fermented beans.

The comparable FQ performance of MCOD and OSD suggests that MCOD replicates the gentle drying conditions of sun drying, allowing pigment breakdown and flavor development to proceed. However, Table 1 also shows a minor occurrence of brown moldy beans (2.00% ± 0.30%) in the MCOD batch—a defect absent in OSD and COD. This may be attributed to humidity fluctuations or limited airflow in the modified oven system. Similar challenges were reported by Díaz et al. (2019), who observed that poor air circulation in semi‐controlled drying setups can support mold growth. Overall, MCOD matched OSD in FQ value, significantly outperforming COD. While the slight mold risk in MCOD warrants further system refinement, its performance underscores its potential as a controlled yet quality‐preserving drying method for cocoa beans.

### 3.3 Effect of Drying Methods on Fat Content and Fatty Acid Levels in Cocoa Beans

Fat content, a key determinant of cocoa bean quality and commercial value (Afoakwa 2010), varied significantly among the drying treatments (*p* < 0.05). As shown in Table 1, beans dried using OSD and MCOD retained the highest fat levels, at 52.82% ± 0.70% and 52.03% ± 0.93%, respectively, whereas COD‐treated beans exhibited a marked reduction in fat content (40.29% ± 2.19%). The control sample showed an intermediate fat level of 49.95% ± 1.21%.

These findings support previous observations that high drying temperatures may promote lipid degradation, reducing the extractable fat fraction (Febrianto et al. 2022). It is likely that thermal stress under COD conditions triggered lipid oxidation or structural modification that interfered with fat extraction efficiency (Bisiw 2018). In contrast, the more gradual drying process in OSD and MCOD may have helped preserve lipid membranes, minimizing oxidative damage and volatilization losses. Higher fat retention in OSD and MCOD is particularly relevant to cocoa processors, as cocoa butter content significantly affects both the yield and quality of chocolate products (Peña‐Correa, Jiménez‐Moreno, and Rodríguez‐Sáenz 2024). The values observed in this study fall within the commercially acceptable range of 45%–55%, with the OSD and MCOD results approaching the upper threshold reported for some improved cocoa cultivars (Dzah et al. 2020; Raghavendra and Prakash 2021).

With respect to FFA content, Table 1 shows that all drying methods resulted in values below the industry rejection threshold of ≥ 1.75% (Peña‐Correa, Jiménez‐Moreno, and Rodríguez‐Sáenz 2024), indicating effective control of lipid hydrolysis. MCOD and OSD yielded nearly identical FFA levels (0.77%), while the control exhibited the lowest value (0.71% ± 0.03%). In contrast, COD‐treated beans recorded the highest FFA concentration at 1.06% ± 0.03%, suggesting that elevated drying temperatures may have accelerated enzymatic or oxidative breakdown of triglycerides (Yang et al. 2018). Notably, the similarity in FFA levels between MCOD and OSD suggests that the modified system successfully prevents excessive lipid degradation, even with its slightly longer drying duration. Moreover, the low FFA content in MCOD‐treated beans also supports the minimal impact of the observed 2.00% mold incidence, as microbial spoilage often coincides with elevated FFA levels (End and Dand 2015; Dzah et al. 2020).

Overall, these findings confirm that OSD and MCOD are more effective than COD in preserving cocoa bean fat quality—both in terms of yield and stability. The data further underscore the importance of temperature control in limiting lipid deterioration during drying.

### 3.4 Effect of Drying Methods on TPC of Cocoa Beans

The TPC of cocoa beans exhibited significant (*ρ* < 0.05) variation across the different drying methods (Table 1), reflecting differences in the oxidative stability of phenolic compounds under varying thermal conditions. Among the dried samples, COD‐treated beans retained the highest TPC (40.55 ± 1.20 mg GAE/g), followed by MCOD (38.80 ± 0.46 mg GAE/g) and OSD (38.13 ± 1.32 mg GAE/g). These values were all lower than that of the undried control (41.63 ± 0.96 mg GAE/g), suggesting that polyphenol oxidation occurred across all drying treatments, albeit to different extents. The relatively high TPC in COD‐treated beans compared to MCOD and OSD may be attributed to the rapid inactivation of PPO and other oxidative enzymes at elevated temperatures (Kadow et al. 2013; Misnawi and Teguh 2010). Heat‐induced enzyme denaturation likely limited polyphenol degradation during drying (Afoakwa 2014; Camu et al. 2008). This protective effect of thermal inactivation has been previously reported by Hii et al. (2009) and Lima et al. (2011), who observed greater polyphenol retention under high‐temperature drying due to early PPO suppression.

However, this apparent advantage came at the expense of FQ, as COD also yielded the lowest FI (0.90 ± 0.04) and the highest proportion of partially fermented beans (10.77% ± 1.75%). These results suggest that while heat may preserve certain phenolics by halting enzymatic action, it may simultaneously impede important biochemical reactions involved in flavor development and pigment (Afoakwa, Kongor, et al. 2013).

On the other hand, OSD‐treated beans—with the lowest TPC—had the best FQ, with only 1.77% ± 0.05% purple beans and the higher FI (1.14 ± 0.02), showing that more extensive oxidation and diffusion of fermentation products occurred under gradual sun drying, consistent with the findings of Misnawi et al. (2004) and ElGamal et al. (2023). Thus, while COD drying improves polyphenol retention by inhibiting enzymatic degradation, it does so at the cost of reduced fermentation efficiency, which is detrimental to the development of cocoa flavor precursors (Payne et al. 2010; Misnawi and Teguh 2010). The MCOD method achieved a middle ground—slightly better preservation of polyphenols than OSD, while still supporting good FQ.

### 3.5 Effect of Drying Methods on Sugar Profile of Cocoa Beans

The sugar composition of cocoa beans—particularly sucrose, glucose, and fructose—is pivotal in the development of flavor precursors during fermentation and drying. In this study, significant variations (*ρ* < 0.05) were observed in the concentrations of these sugars among the different drying methods applied to fermented beans (Table 1).

Sucrose content decreased from 3.61 ± 0.12 mg/g in the control (undried) sample to 3.20 ± 0.16 mg/g in the MCOD‐treated beans. Similarly, glucose levels declined significantly from 9.17 ± 0.23 mg/g in the control to 7.85 ± 0.20 mg/g in COD, while fructose followed the same trend, decreasing from 10.11 ± 0.18 mg/g in the control to 8.31 ± 0.29 mg/g in COD. These reductions are attributed to the degradation of sugars through thermal reactions such as the Maillard reaction and caramelization, which are intensified under higher drying temperatures (*ρ* < 0.05) (Redgwell et al. 2003).

Among the treatments, Table 1 shows that OSD retained relatively higher concentrations of reducing sugars (glucose: 8.70 ± 0.26 mg/g, fructose: 9.26 ± 0.25 mg/g) compared to COD and MCOD. This could be due to the milder and fluctuating temperature conditions during OSD, which mitigated thermal degradation (Albak and Tekin 2016). In contrast, the COD process, characterized by higher and more uniform temperatures, resulted in greater (*ρ* < 0.05) sugar loss, aligning with previous findings that elevated drying temperatures accelerate sugar degradation (Redgwell et al. 2003). Interestingly, MCOD, designed to mimic OSD conditions, exhibited slightly lower glucose (8.41 ± 0.24 mg/g) and fructose (8.96 ± 0.23 mg/g) levels compared to OSD. This discrepancy may be attributed to microbial metabolism of sugars, as indicated by the higher incidence of moldy beans (2.00 ± 0.30) in the MCOD batch. The presence of mold can lead to additional sugar consumption during drying, resulting in reduced residual sugar levels (Hart 2019).

Since reducing sugars (glucose and fructose) serve as essential substrates in the Maillard reaction during roasting, their retention is essential for flavor development in cocoa. These findings highlight the importance of optimizing drying conditions to preserve key sugars without encouraging microbial spoilage.

### 3.6 Effect of Drying Methods on Organic Acid Content of Cocoa Beans

Organic acids play critical roles in flavor development and microbial ecology during cocoa fermentation and post‐processing (Afoakwa, Quao, et al. 2013). This study assessed how drying methods influenced the levels of key organic acids—acetic acid, lactic acid, and citric acid—in fermented cocoa beans.

As shown in Table 1, acetic acid—the dominant acid produced by acetic acid bacteria during fermentation—was significantly reduced by all drying methods (*ρ* < 0.05). The control recorded the highest level (5.82 ± 0.24 mg/g), while the lowest was observed in COD‐treated beans (3.94 ± 0.16 mg/g). OSD and MCOD retained slightly higher levels (4.53 ± 0.15 and 4.27 ± 0.18 mg/g, respectively). These reductions are attributed to the volatile nature of acetic acid and its rapid loss under heat, especially in COD (Jinap et al. 2016). COD's high temperature likely enhanced evaporation, reducing acid retention (Camu et al. 2008; Afoakwa 2014). The milder losses in OSD and MCOD suggest slower evaporation under ambient or modified thermal conditions, preserving more acid content.

Lactic acid, a less volatile organic acid, was comparatively more stable across treatments. As shown in Table 1, COD still showed a slight but significant decrease (1.72 ± 0.10 mg/g) compared to the control (2.11 ± 0.08 mg/g) and OSD (2.01 ± 0.09 mg/g). The lower values in COD and MCOD (1.88 ± 0.11) may reflect partial degradation of lactic acid under heat and diminished post‐fermentation microbial activity (Lima et al. 2011).

Interestingly, as shown in Table 1, citric acid levels did not differ significantly across the drying treatments (*ρ* > 0.05). This acid is generally less reactive and more stable under mild to moderate heat conditions. The control sample retained the highest level (0.75 ± 0.05 mg/g) of citric acid, with significant reductions observed in all dried samples: OSD (0.60 ± 0.11 mg/g), MCOD (0.58 ± 0.13 mg/g), and COD (0.43 ± 0.03 mg/g). These losses are possibly due to microbial metabolism during fermentation and further degradation during thermal drying. COD resulted in the greatest decline, likely due to enzyme inactivation and citric acid thermal degradation.

Overall, drying methods significantly influenced the organic acid profile of cocoa beans (*ρ* < 0.05), with COD causing the most pronounced reduction due to elevated drying temperatures and enhanced volatilization. The moderate acid retention in OSD supports its traditional use in preserving flavor precursors, while the MCOD approach, though mimicking OSD conditions, showed more acid degradation—possibly due to extended drying time and microbial deterioration, as corroborated by its cut‐test score.

### 3.7 Effect of Drying Methods on Amino Acid Profile of Cocoa Beans

The results (Table 2) demonstrate that drying methods significantly influenced the free amino acid profile of cocoa beans. Among the treatments, OSD and MCOD resulted in notably (*p* < 0.05) higher concentrations of most amino acid groups compared to COD and the control.

**TABLE 2:** Free amino acid composition of cocoa beans under different drying treatments.

Quality parameter	Control	Drying treatment		
		OSD	COD	MCOD
Free amino acids (mg/g)				
Acidic amino acid	4.36^a^ ± 0.11	3.92^b^ ± 0.18	3.37^b^ ± 0.20	3.77^b^ ± 0.24
Aspartate (Asp)	0.49^a^ ± 0.02	0.45^b^ ± 0.04	0.35^c^ ± 0.03	0.43^b^ ± 0.04
Asparagine (Asn)	1.19^a^ ± 0.04	1.07^b^ ± 0.05	0.96^bc^ ± 0.08	1.05^b^ ± 0.06
Glutamate (Glu)	1.74^a^ ± 0.04	1.57^b^ ± 0.07	1.48^c^ ± 0.06	1.56^b^ ± 0.11
Glutamine (Gln)	0.94^a^ ± 0.01	0.83^b^ ± 0.02	0.58^d^ ± 0.03	0.73^c^ ± 0.03
Basic amino acids	3.36^a^ ± 0.06	2.65^b^ ± 0.11	0.32^c^ ± 0.17	2.39^b^ ± 0.11
Arginine (Arg)	1.41^a^ ± 0.02	1.25^b^ ± 0.02	0.10^d^ ± 0.05	1.08^c^ ± 0.02
Histidine (His)	0.72^a^ ± 0.01	0.48^b^ ± 0.03	0.14^c^ ± 0.03	0.42^bc^ ± 0.04
Lysine (Lys)	1.23^a^ ± 0.03	0.92^b^ ± 0.06	0.08^c^ ± 0.09	0.89^b^ ± 0.05
Hydrophobic amino acids	8.20^a^ ± 0.20	6.14^b^ ± 0.24	3.20^c^ ± 0.29	6.18^b^ ± 0.34
Alanine (Ala)	1.21^a^ ± 0.03	1.07^b^ ± 0.02	0.19^c^ ± 0.08	1.10^b^ ± 0.02
Leucine (Leu)	2.28^a^ ± 0.06	1.68^b^ ± 0.03	0.87^c^ ± 0.07	1.65^b^ ± 0.06
Isoleucine (Ile)	1.41^a^ ± 0.04	1.21^b^ ± 0.09	1.02^c^ ± 0.04	1.25^b^ ± 0.08
Tyrosine (Tyr)	1.22^a^ ± 0.03	0.93^b^ ± 0.03	0.55^c^ ± 0.07	0.88^bc^ ± 0.10
Phenylalanine (Phe)	1.02^a^ ± 0.01	0.45^b^ ± 0.03	0.21^c^ ± 0.01	0.47^b^ ± 0.04
Valine (Val)	1.06^a^ ± 0.03	0.80^b^ ± 0.04	0.36^c^ ± 0.02	0.83^b^ ± 0.04
Other amino acids	2.33^a^ ± 0.11	1.94^b^ ± 0.14	1.56^a^ ± 0.19	1.83^b^ ± 0.17
Glycine (Gly)	0.33^a^ ± 0.01	0.26^b^ ± 0.04	0.17^d^ ± 0.03	0.21^c^ ± 0.03
Tryptophane (Trp)	0.28^a^ ± 0.02	0.23^b^ ± 0.02	0.17^c^ ± 0.01	0.21^b^ ± 0.04
Threonine (Thr)	0.47^a^ ± 0.02	0.42^b^ ± 0.03	0.33^c^ ± 0.02	0.40^b^ ± 0.03
Serine (Ser)	0.58^a^ ± 0.02	0.52^b^ ± 0.01	0.47^c^ ± 0.04	0.51^b^ ± 0.02
Proline (Pro)	0.52^a^ ± 0.03	0.41^b^ ± 0.02	0.37^c^ ± 0.07	0.42^b^ ± 0.03
Methionine (Met)	0.15^a^ ± 0.01	0.10^a^ ± 0.02	0.05^b^ ± 0.02	0.08^ab^ ± 0.02
Total amino acid	18.25^a^ ± 0.48	14.65^b^ ± 0.67	8.45^c^ ± 0.85	14.17^b^ ± 0.86

*Note:* Means in a row with the same superscript letter are not statistically significant (*p* ≤ 0.05; Tukey's Post Hoc Test). Control = Fermented undried cocoa.

Abbreviations: COD, convective oven drying; Control, Fermented undried cocoa; MCOD, modified convective oven drying; OSD, open sun drying.

Acidic amino acids (aspartate, glutamate, asparagine, and glutamine) were highest in the control group (4.36 ± 0.11 mg/g) and lowest in COD‐treated beans (3.37 ± 0.20 mg/g). However, the total acidic amino acid content in OSD (3.92 ± 0.18 mg/g) and MCOD (3.77 ± 0.24 mg/g) was relatively higher than COD, though the differences were not statistically significant (*p* > 0.05). The higher retention of acidic amino acids in OSD and MCOD may be attributed to slower drying rates at lower temperatures, which minimized thermal degradation. This observation is supported by Norasmadi et al. (2024), who reported that gradual drying under mild temperatures preserves amino acid integrity. Conversely, COD likely led to greater thermal degradation and amino acid loss due to accelerated Maillard reactions at higher temperatures (Peña‐Correa, Ataç Mogol, and Fogliano 2024).

Basic amino acids, particularly histidine, arginine, and lysine, were also significantly affected by drying methods (Table 2). OSD (2.65 ± 0.11 mg/g) and MCOD (2.39 ± 0.11 mg/g) recorded significantly (*p* < 0.05) higher levels than COD (0.32 ± 0.17 mg/g). Arginine content, a critical flavor precursor, was significantly (*p* < 0.05) higher in OSD (1.25 ± 0.02 mg/g) and MCOD (1.08 ± 0.02 mg/g) than in COD (0.10 ± 0.05 mg/g), reflecting improved amino acid preservation under milder drying conditions. Lysine levels followed a similar trend, with higher concentrations in OSD (0.92 ± 0.06 mg/g) and MCOD (0.89 ± 0.05 mg/g) than COD (0.08 ± 0.09 mg/g). This supports findings by Nath et al. (2022) that lysine is highly susceptible to degradation under high‐temperature drying due to non‐enzymatic browning reactions.

As shown in Table 2, hydrophobic amino acids—which contribute significantly to cocoa flavor—also varied among treatments. Among the treatment samples, the highest total hydrophobic amino acid content was found in OSD‐treated beans (6.14 ± 0.24 mg/g), followed by MCOD‐treated beans (6.18 ± 0.34 mg/g), with COD recording the lowest value (3.20 ± 0.29 mg/g). OSD‐treated beans showed significantly (*p* < 0.05) higher concentrations of leucine (1.68 ± 0.03 mg/g), isoleucine (1.21 ± 0.09 mg/g), and valine (0.80 ± 0.04 mg/g) compared to COD. These amino acids serve as critical precursors for pyrazine and aldehyde formation during roasting, and their higher concentrations in OSD and MCOD treatments suggest better retention due to the lower thermal impact (Rottiers et al. 2019; Pereira et al. 2022).

Regarding other amino acids, Table 2 shows that the total content was highest in the control (2.33 ± 0.11 mg/g), with OSD (1.94 ± 0.14 mg/g) and MCOD (1.83 ± 0.17 mg/g) showing moderate levels, and COD exhibiting the lowest (1.56 ± 0.19 mg/g). While serine and tryptophan did not show statistically significant (*p* > 0.05) variation across treatments, proline levels were significantly (*p* < 0.05) higher in OSD (0.41 ± 0.02 mg/g) and MCOD (0.42 ± 0.03 mg/g) than COD (0.37 ± 0.07 mg/g). This finding indicates that moderate‐temperature drying favors the retention of proline, which is known for its role in roasted, nutty, and caramel‐like flavor development and stress response mechanisms in cocoa beans (Santander Munoz et al. 2020). Methionine content remained relatively stable across treatments, reflecting its greater resistance to thermal degradation.

Overall, total free amino acid content was highest (*p* < 0.05) in the control (18.25 ± 0.48 mg/g), followed by OSD (14.65 ± 0.67 mg/g) and MCOD (14.17 ± 0.86 mg/g), while COD showed the lowest value (8.45 ± 0.85 mg/g). These results highlight that drying at lower temperatures (as in OSD and MCOD) enhances amino acid retention in cocoa beans, whereas higher temperatures used in COD promote degradation through non‐enzymatic browning and Maillard reactions. These findings align with previous literature emphasizing the influence of drying conditions on cocoa bean biochemical composition and potential flavor development (Jinap et al. 2016; Nath et al. 2022).

### 3.8 Effect of Drying Methods on Volatile Organic Compound (VOC) Profile of Cocoa Beans

Drying treatments significantly influenced the profile and concentration of VOCs in cocoa beans, which are critical contributors to flavor and aroma quality. As shown in Table 3, a total of 37 VOCs were identified in the cocoa bean samples, comprising key chemical groups such as aldehydes (8), ketones (8), alcohols (6), esters (6), and pyrazines (9). Each of these groups contributes distinct aroma characteristics to cocoa, enhancing its overall sensory profile (Jinap et al. 1995; Afoakwa et al. 2008).

**TABLE 3:** Volatile organic compound profiles of cocoa beans under different drying treatments.

Parameter	Odor description	Control	Drying treatment		
			OSD	COD	MCOD
Volatile organic compounds (mg/kg)					
Aldehydes		1.69^a^ ± 0.03	1.47^b^ ± 0.06	0.90^c^ ± 0.07	1.45^b^ ± 0.04
2‐Methyl butanal	Cocoa, chocolate	0.21^a^ ± 0.00	0.13^a^ ± 0.00	0.20^a^ ± 0.00	0.13^a^ ± 0.00
3‐methyl butanal	Cocoa, chocolate	0.29^a^ ± 0.01	0.38^a^ ± 0.02	0.11^ab^ ± 0.02	0.39^a^ ± 0.01
Acetaldehyde	Bitter, pungent	0.57^a^ ± 0.01	0.02^b^ ± 0.00	0.04^b^ ± 0.01	0.01^b^ ± 0.01
Benzaldehyde	Sweet, fruity	0.19^a^ ± 0.00	0.62^b^ ± 0.02	0.37^c^ ± 0.01	0.60^b^ ± 0.01
Phenylacetaldehyde	Floral, sweet	0.31^a^ ± 0.01	0.23^a^ ± 0.02	0.12^ab^ ± 0.02	0.23^a^ ± 0.01
2‐Phenyl‐2‐butenal	Sweet, cinnamon	0.05^a^ ± 0.00	0.04^a^ ± 0.00	0.03^a^ ± 0.01	0.04^a^ ± 0.00
5‐Methyl‐2‐phenyl‐2‐hexanal	Cocoa, chocolate	0.03^a^ ± 0.00	0.02^a^ ± 0.00	0.02^a^ ± 0.00	0.02^a^ ± 0.00
5‐phenyl‐2‐methylpentanal	Floral	0.04^a^ ± 0.00	0.03^a^ ± 0.00	0.01^a^ ± 0.00	0.03^a^ ± 0.00
Ketones		0.14^a^ ± 0.00	1.59^b^ ± 0.12	0.90^c^ ± 0.20	1.50^b^ ± 0.10
2‐Nonanone	Fruity	0.02^a^ ± 0.00	0.09^a^ ± 0.01	0.05^a^ ± 0.00	0.08^a^ ± 0.01
2‐Pentanone	Fruity	0.03^a^ ± 0.00	0.13^a^ ± 0.00	0.05^a^ ± 0.03	0.13^a^ ± 0.00
3‐Hydroxy‐2‐butanone (acetoin)	Buttery, creamy	0.01^a^ ± 0.00	0.18^a^ ± 0.02	0.08^a^ ± 0.05	0.18^a^ ± 0.01
1‐Hydroxy‐2‐propanone	Sweet, caramel	0.00^a^ ± 0.00	0.19^b^ ± 0.00	0.20^c^ ± 0.01	0.16^abc^ ± 0.06
2,3‐Pentanedione	Bitter, pungent	0.02^a^ ± 0.00	0.10^a^ ± 0.00	0.05^a^ ± 0.01	0.10^a^ ± 0.00
3‐Hydroxy‐2‐pentanone	Buttery, creamy	0.02^a^ ± 0.00	0.32^b^ ± 0.02	0.14^ab^ ± 0.02	0.31^b^ ± 0.01
2‐Heptanone	Fruity	0.02^a^ ± 0.00	0.25^b^ ± 0.06	0.16^ab^ ± 0.04	0.22^b^ ± 0.00
Acetophenone	Floral, sweet	0.02^a^ ± 0.00	0.33^b^ ± 0.01	0.17^ab^ ± 0.04	0.32^b^ ± 0.01
Alcohols		6.12^a^ ± 0.09	5.15^b^ ± 0.26	2.26^c^ ± 0.42	4.90^b^ ± 0.44
2‐Methyl‐1‐ propanol	Winey, ethereal	1.07^a^ ± 0.03	0.28^b^ ± 0.04	0.21^b^ ± 0.01	0.25^b^ ± 0.02
2,3‐Butanediol	Fruity, buttery	0.89^a^ ± 0.01	1.84^b^ ± 0.18	1.27^b^ ± 0.26	1.74^b^ ± 0.34
2‐Pentanol	Fruity, sweet	0.27^a^ ± 0.01	0.11^a^ ± 0.01	0.07^ab^ ± 0.01	0.13^a^ ± 0.02
Benzyl alcohol	Floral, sweet	0.31^a^ ± 0.00	0.28^a^ ± 0.05	0.18^ab^ ± 0.03	0.28^b^ ± 0.03
3‐Methyl butanol	Fruity	0.51^a^ ± 0.01	0.53^a^ ± 0.04	0.33^c^ ± 0.10	0.47^ab^ ± 0.02
2‐Phenylethanol	Floral	3.07^a^ ± 0.03	2.11^b^ ± 0.04	0.23^c^ ± 0.01	2.03^b^ ± 0.01
Esters		2.42^a^ ± 0.03	0.59^b^ ± 0.11	0.23^c^ ± 0.02	0.65^b^ ± 0.07
Isoamyl acetate	Fruity	1.48^a^ ± 0.03	0.02^b^ ± 0.01	N/A	0.01^b^ ± 0.00
Ethyl acetate	Fruity	0.17^a^ ± 0.00	0.45^b^ ± 0.06	0.20^a^ ± 0.01	0.49^b^ ± 0.04
Methyl acetate	Fruity	0.12^a^ ± 0.00	0.01^a^ ± 0.00	N/A	0.01^a^ ± 0.00
Isobutyl acetate	Fruity	0.12^a^ ± 0.00	0.03^a^ ± 0.02	N/A	0.03^a^ ± 0.01
2‐Pentyl acetate	Fruity	0.21^a^ ± 0.00	0.04^a^ ± 0.01	0.03^ab^ ± 0.01	0.04^a^ ± 0.01
2‐Phenyl ethyl acetate	Floral	0.32^a^ ± 0.00	0.04^b^ ± 0.01	N/A	0.03^b^ ± 0.01
Pyrazines		N/A	8.66^a^ ± 0.44	3.40^b^ ± 0.62	8.63^a^ ± 0.48
Methylpyrazine	Cocoa, chocolate	N/A	0.14^a^ ± 0.00	0.13^a^ ± 0.00	0.13^a^ ± 0.00
2,3‐Dimethyl pyrazine	Chocolate, cocoa	N/A	0.50^a^ ± 0.03	0.39^a^ ± 0.05	0.51^a^ ± 0.01
2,5‐Dimethyl pyrazine	Chocolate, nutty	N/A	0.17^a^ ± 0.01	0.04^a^ ± 0.01	0.16^a^ ± 0.02
2,6‐Dimethyl pyrazine	Chocolate, nutty	N/A	0.12^a^ ± 0.00	0.01^a^ ± 0.00	0.12^a^ ± 0.00
2‐Ethyl‐6‐methyl pyrazine	Cocoa, chocolate	N/A	0.07^a^ ± 0.00	0.02^a^ ± 0.01	0.09^a^ ± 0.01
2,3‐Dimethyl‐5‐ethylpyrazine	Chocolate, nutty	N/A	0.33^a^ ± 0.01	0.01^b^ ± 0.00	0.33^a^ ± 0.02
Trimethyl pyrazine	Chocolate, cocoa	N/A	2.16^a^ ± 0.18	0.52^b^ ± 0.16	2.14^a^ ± 0.12
Tetramethyl pyrazine	Chocolate, nutty	N/A	5.11^a^ ± 0.19	2.26^b^ ± 0.38	5.07^a^ ± 0.29
3,5‐Diethyl‐2‐methyl pyrazine	Cocoa, chocolate	N/A	0.06^a^ ± 0.02	0.02^a^ ± 0.01	0.08^a^ ± 0.01

*Note:* Means in a row with the same superscript letter are not statistically significant (*p* ≤ 0.05; Tukey's Post Hoc Test). Odor description from Rodriguez‐Campos et al. (2011), Hinneh et al. (2018), Rottiers et al. (2019), and Kone et al. (2020).

Abbreviations: COD, convective oven drying; Control, Fermented undried cocoa; MCOD, modified convective oven drying; OSD, open sun drying.

Aldehydes, particularly those generated through Strecker degradation and lipid oxidation, were found to be prominent volatile compounds across all drying treatments (Table 3). The control sample recorded the highest total aldehyde concentration (1.69 ± 0.03 mg/kg), followed by OSD (1.47 ± 0.06 mg/kg) and MCOD (1.45 ± 0.04 mg/kg), while COD yielded the lowest levels (0.90 ± 0.07 mg/kg), suggesting that high‐temperature convective drying promotes the thermal loss or degradation of aldehydes (Ho et al. 2014). Importantly, the elevated concentrations of leucine, isoleucine, valine, and phenylalanine observed in the FAA profiles under OSD and MCOD (Table 2) closely correspond with the higher levels of 2‐methylbutanal, 3‐methylbutanal, and benzaldehyde in the associated VOC data (Table 3). These volatile aldehydes are characteristic products of the Strecker degradation pathway, arising from the decarboxylation and deamination of α‐amino acids during thermal processing (Counet et al. 2002; Aprotosoaie et al. 2015). This biochemical link substantiates the role of amino acid abundance, particularly under moderate drying conditions, in enhancing the aromatic potential of cocoa beans. Acetaldehyde, which contributes a sharp, bitter aroma, was notably reduced in COD, likely due to its high volatility and susceptibility to thermal degradation at elevated temperatures. In contrast, desirable aldehydes such as benzaldehyde and 2‐methylbutanal—associated with sweet, fruity, and cocoa‐like sensory attributes—were better preserved in OSD and MCOD treatments. This observation highlights the effectiveness of moderate‐temperature drying in preserving key aroma‐active compounds and maintaining the aromatic integrity of cocoa beans (Niemenak et al. 2006).

Ketones, which are responsible for imparting sweet, caramel, and buttery flavors, were generally elevated under OSD and MCOD. The highest total concentrations of ketones were observed in beans subjected to OSD (1.59 ± 0.12 mg/kg) and MCOD (1.50 ± 0.10 mg/kg), significantly (*p* < 0.05) higher than those in the control (0.14 ± 0.07 mg/kg) and COD (0.90 ± 0.20 mg/kg). These increases suggest enhanced formation of Maillard reaction products under moderate drying conditions, consistent with findings by Counet et al. (2002) and Erazo Solorzano et al. (2023). Ketones are typically formed through a deamination reaction involving amino acids such as threonine and serine, which yield α‐keto acids that subsequently undergo further reduction (Aprotosoaie et al. 2015). Notably, 1‐hydroxy‐2‐propanone (hydroxyacetone), a Maillard‐derived ketone absent in the control sample, was detected in all drying treatments, with the highest concentration recorded in COD (0.20 mg/kg). Similarly, desirable ketones such as 3‐hydroxy‐2‐butanone (acetoin), 2‐pentanone, and acetophenone—which are characterized by low odor‐activity thresholds and contribute buttery, fruity, and floral notes—were more abundant under moderate drying treatments. This further highlights the temperature sensitivity of ketone formation and retention during cocoa bean drying (Schwan and Wheals 2004).

Alcohols constituted a significant fraction of the VOCs detected in all cocoa samples, with notable variations across drying methods (Table 3). Among the treated samples, the highest total alcohol content was recorded in OSD beans (5.15 ± 0.26 mg/kg), followed by MCOD at 4.90 ± 0.44 mg/kg and COD at 2.26 ± 0.42 mg/kg. The control sample recorded the highest overall alcohol concentration at 6.12 ± 0.09 mg/kg, indicating that drying generally leads to a reduction in volatile alcohols, albeit at different rates depending on the drying conditions. Among the individual alcohols, 2‐methyl‐1‐propanol, associated with undesirable winey and ethereal aromas (Rottiers et al. 2019), had the highest concentration in the control (1.07 ± 0.03 mg/kg) and decreased progressively across OSD (0.28 ± 0.04 mg/kg), MCOD (0.25 ± 0.02 mg/kg), and COD (0.21 ± 0.01 mg/kg). This alcohol is primarily produced through yeast fermentation and is highly volatile, making it susceptible to evaporation during high‐temperature drying (Erazo Solorzano et al. 2023). 2‐Pentanol, which imparts a fruity and sweet aroma, followed a similar trend: control (0.27 ± 0.01 mg/kg) > OSD (0.11 ± 0.01 mg/kg) > MCOD (0.13 ± 0.02 mg/kg) > COD (0.07 ± 0.01 mg/kg). Its relative stability under MCOD and OSD suggests milder thermal stress compared to the more intense conditions in COD. 3‐Methylbutanol, another fruity alcohol that contributes to sweet, banana‐like notes, was retained best in OSD (0.53 ± 0.01 mg/kg) and MCOD (0.47 ± 0.02 mg/kg), compared to COD (0.33 ± 0.10 mg/kg), supporting previous findings (Albak and Tekin 2016; Kone et al. 2020). Other alcohols, such as 3‐butenol, benzyl alcohol, and 2‐phenylethanol, all known for their floral and fruity characteristics, followed similar patterns across treatments. Notably, 2,3‐butanediol, a compound contributing to creamy and fruity aromas, showed higher concentrations in all dried samples (1.27–1.84 mg/kg) compared to the control (0.89 ± 0.01 mg/kg). Its levels were especially elevated in OSD (1.84 ± 0.18 mg/kg) and MCOD (1.74 ± 0.34 mg/kg). This increase suggests either the formation of 2,3‐butanediol during drying—potentially from precursors such as acetoin—or microbial activity during extended or slower drying phases. Certain bacteria and fungi, including *Bacillus* spp., *Klebsiella, and Saccharomyces cerevisiae*, are known to produce 2,3‐butanediol during sugar metabolism under fermentation‐like conditions (Ji et al. 2011; Celińska and Grajek 2009). OSD, which is more susceptible to environmental exposure, may encourage such microbial proliferation, especially under humid or unhygienic conditions (Jinap et al. 2010). While moderate levels of 2,3‐butanediol may contribute positively to cocoa aroma, its elevated presence may also signal post‐fermentation microbial activity or the onset of spoilage, depending on drying hygiene and control (De Vuyst and Weckx 2016; Hinneh et al. 2018).

Esters, which are key contributors to the fruity and floral aroma of cocoa beans, were significantly influenced by the drying method employed (Table 3). Among all the treatments, the control beans recorded the highest total ester concentration (2.42 ± 0.03 mg/kg), followed by MCOD (0.65 ± 0.07 mg/kg), OSD (0.59 ± 0.11 mg/kg), and the lowest in COD (0.23 ± 0.02 mg/kg). The significant reduction in esters following drying (*p* < 0.05) suggests that many ester compounds are either thermally unstable or highly volatile, and hence susceptible to evaporation or degradation under drying conditions. Among the individual esters, isoamyl acetate, known for its intense fruity and banana‐like aroma, was the most abundant in the control sample (1.48 ± 0.03 mg/kg) but was drastically reduced in all dried samples, especially in OSD (0.02 ± 0.01 mg/kg), MCOD (0.01 ± 0.00 mg/kg), and COD (not detected). This dramatic decline aligns with the compound's low boiling point and high volatility, making it particularly vulnerable during open‐sun exposure and higher‐temperature convective drying. Similarly, 2‐phenylethyl acetate, which imparts a desirable floral note to cocoa (e.g., a rose or honey‐like aroma), also exhibited substantial reductions in concentration after drying: from 0.32 ± 0.00 mg/kg in the control to as low as 0.03 ± 0.01 mg/kg in MCOD and 0.04 ± 0.01 mg/kg in COD. Ethyl acetate, another fruity ester, interestingly showed slightly higher concentrations in OSD (0.45 ± 0.06 mg/kg) and MCOD (0.49 ± 0.04 mg/kg) than in the control (0.17 ± 0.00 mg/kg), suggesting possible in situ formation during drying. This may be attributed to enzymatic or microbial esterification reactions involving ethanol and acetic acid, particularly under moist conditions early in the drying process when microbial activity may persist (De Vuyst and Weckx 2016; Hue et al. 2016). The same trend was noted, albeit less markedly, in methyl acetate, which was retained only in trace amounts (≤ 0.12 mg/kg), with no significant variation across treatments except absence in COD. Notably, the ester profile suggests that drying, especially COD drying, significantly reduces the concentration of aroma‐contributing esters, likely due to elevated temperatures and oxygen, which promote oxidation, hydrolysis, or volatilization of these compounds (Bikomo et al. 2016). The better retention observed in MCOD may be attributed to the milder and more controlled drying environment, which minimizes losses while still allowing some microbial‐driven biosynthesis of certain esters.

Pyrazines—an important group of nitrogen‐containing heterocyclic compounds responsible for roasted, nutty, and cocoa‐like aromas in chocolate—showed some of the most pronounced variations in VOC profiles across the different drying methods (Table 3). While these compounds are predominantly formed through Maillard reactions during roasting generation (Jinap et al. 2010; Rodriguez‐Campos et al. 2011), the preservation of their precursors during the drying stage is essential for their subsequent development and for enhancing the overall flavor profile of cocoa products (Meng et al. 2025). In this study, OSD yielded the highest total pyrazine concentration (8.66 ± 0.44 mg/kg), closely followed by MCOD (8.63 ± 0.48 mg/kg). In contrast, conventional COD resulted in a significantly lower total pyrazine content (3.40 ± 0.62 mg/kg) (*p* < 0.05). These results are striking, suggesting that slower and more natural drying processes such as OSD and MCOD may be more favorable for preserving amino acids and other precursors essential for pyrazine formation during subsequent roasting. Among individual pyrazines, trimethyl pyrazine and tetramethyl pyrazine—known contributors to characteristic chocolate, nutty, and roasted notes—were most abundant in OSD (2.16 ± 0.18 mg/kg and 5.11 ± 0.19 mg/kg, respectively) and MCOD (2.14 ± 0.12 mg/kg and 5.07 ± 0.29 mg/kg), but showed significantly reduced levels in COD (0.52 ± 0.16 mg/kg and 2.26 ± 0.38 mg/kg, respectively). Similarly, 2,3‐dimethyl‐5‐ethylpyrazine, 2,3‐dimethyl pyrazine, and 2,5‐dimethyl pyrazine followed the same trend, showing markedly higher concentrations in OSD and MCOD compared to COD. This pattern aligns with the findings of Yu et al. (2021), who reported that excessive drying temperatures can prematurely trigger Maillard reactions during drying itself, leading to early pyrazine formation and volatilization losses, thereby reducing the pool of available precursors for further pyrazine synthesis during roasting. Furthermore, the relatively lower temperature and slower drying kinetics in OSD and MCOD may have preserved more nitrogenous precursors and reducing sugars, offering a more favorable chemical environment for pyrazine formation post‐drying. The results also suggest that rapid drying in COD may disrupt nitrogen metabolism, possibly through thermal denaturation of key enzymes or amino acid degradation, thereby limiting pyrazine biosynthesis pathways. Therefore, while COD may offer speed and microbial safety advantages, it may also compromise flavor precursor retention—a trade‐off that must be carefully considered in post‐harvest cocoa processing.

These findings underscore the critical role of drying method selection in influencing flavor quality in cocoa beans, particularly with respect to key aroma‐active compounds such as pyrazines. For premium chocolate production where flavor is paramount, gentler drying methods like OSD and MCOD may be more suitable for preserving the complex precursor chemistry needed to develop rich, roasted cocoa flavors during roasting.

### 3.9 PCA of Post‐Harvest Cocoa Quality Attributes

**FIGURE 2:**
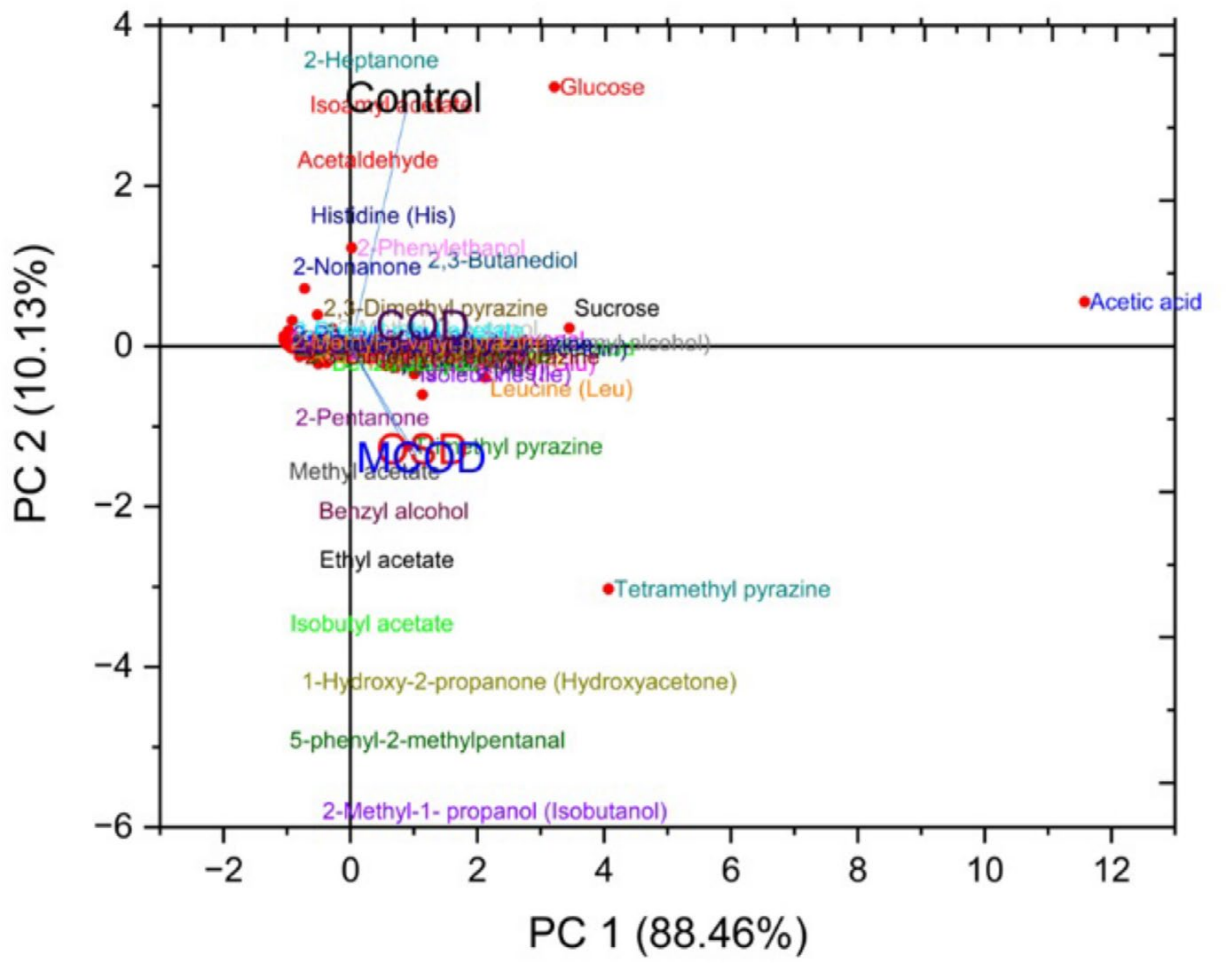
Biplot of post‐harvest quality attributes of cocoa beans under different drying treatments. COD, convective oven drying; MCOD, modified convective oven drying; OSD, open sun drying.

To evaluate the collective impact of different drying treatments on cocoa bean quality, PCA was employed. This multivariate approach allowed for the simultaneous assessment of VOCs, precursors, and organic acid content, thereby identifying the most influential variables and clustering patterns across treatments. The PCA biplot (Figure 2) visually represents the distribution of quality‐related metabolites and their associations with each drying method.

The first two principal components (PC1 and PC2) explain a cumulative variance of 98.59%, with PC1 accounting for 88.46% and PC2 contributing 10.13%. This high cumulative variance underscores the robustness of the PCA in capturing the underlying chemical variations induced by the different drying regimes. The control group is distinctly separated along the positive PC2 axis, showing strong associations with glucose, hydroxyacetone, histidine, and 2‐nonanone. This suggests a higher retention of reducing sugars, amino acids, and certain volatiles in the absence of thermal processing. The positioning reinforces literature findings that thermal drying induces substantial biochemical transformations, including Maillard reactions and the loss of low molecular weight compounds through volatilization (Afoakwa et al. 2008; Herrera‐Rocha et al. 2021).

Conversely, the COD treatment is markedly separated along the positive PC1 axis and strongly associated with acetic acid and methylpyrazine. These associations indicate a pronounced influence of COD on Maillard reaction pathways, promoting the formation of nitrogenous aroma compounds such as pyrazines, which are essential contributors to chocolate's roasted and nutty aroma profile (Quelal et al. 2023). The high loading of acetic acid further suggests elevated volatilization due to the rapid and high‐temperature drying environment (Pereira et al. 2022). However, COD shows a pronounced negative loading on PC2, indicating substantial losses in amino acids and sugars—key precursors for aroma development—which aligns with prior studies on the effects of intense drying on cocoa quality (Nath et al. 2022; Hu et al. 2024). The MCOD treatment occupies an intermediate space between COD and OSD along PC1 while also showing less negative loading on PC2. MCOD's association with methylpyrazine and lactic acid reflects a more balanced drying process that favors both the generation of Maillard‐derived aroma compounds and the retention of important fermentation metabolites like 2,3‐butanediol. This suggests that MCOD may offer a compromise between flavor enhancement and precursor preservation, possibly due to modifications in temperature or drying duration that reduce thermal degradation (Viesser et al. 2021). The OSD treatment, positioned near MCOD on PC1 but with more negative PC2 loading, indicates moderate influence on cocoa chemistry. Its proximity to compounds such as 2‐methyl butanal and isobutanol reflects the preservation of key Strecker degradation products and fermentation‐derived alcohols. Moreover, the strong association with pyrazines suggests that the lower temperatures of sun drying are sufficient to drive extensive Maillard reactions, in line with reports of milder flavor development in sun‐dried cocoa (Erazo Solorzano et al. 2023; Ullrich 2024).

The PCA clearly delineates how post‐harvest drying strategies significantly influence the chemical composition of cocoa beans. Notably, both OSD and MCOD preserve a greater proportion of fermentation‐derived precursors and enhance the formation of aldehydes, pyrazines, and other Maillard‐related volatiles, which are critical to flavor development. In contrast, COD retains more undesirable organic acids while showing reduced intensity in desirable flavor compounds. These findings underscore the pivotal role of drying protocols in shaping cocoa quality attributes. Importantly, the similarity between MCOD and OSD suggests that MCOD can serve as a viable alternative to traditional sun drying, offering a controlled approach for optimizing post‐harvest processes toward specific flavor profiles.

## 4 Conclusions

This study evaluated the effects of different drying techniques—OSD, conventional oven drying (COD), and MCOD—on the post‐harvest quality of fermented Ghanaian cocoa beans. The findings indicate that MCOD effectively simulates the thermal and moisture conditions of OSD, producing beans with comparable FI, fat content, amino acid profiles, and key aroma‐active volatile compounds (*p* > 0.05). In contrast, COD‐treated beans showed significant deviations in these quality attributes, underscoring the limitations of using fixed‐condition dryers in replicating natural drying behavior. The chemometric evaluation using PCA further reinforced the similarity between OSD and MCOD, while clearly separating COD based on its distinct volatile and chemical profiles.

The MCOD system, by leveraging real‐time environmental profiles and gradual diurnal control of temperature and relative humidity, presents a reliable and reproducible alternative to traditional sun drying, particularly in regions affected by inconsistent weather or limited access to drying infrastructure. By improving drying efficiency and product uniformity and reducing dependency on weather conditions, MCOD also supports more sustainable post‐harvest practices for smallholder farmers and larger processing operations alike.

While promising, further optimization is recommended, including the integration of staged humidity control, air velocity management, and potential hybrid designs to enhance simulation accuracy. Future studies should also assess the impact of MCOD‐dried beans on downstream processing parameters such as conching, tempering, fat bloom development, and the stability of flavor and polyphenolic compounds in finished cocoa products. Comparative trials using different cocoa genotypes are also needed to validate the broader applicability of the MCOD approach.

## Author Contributions

**Lukeman Haruna:** conceptualization (lead), data curation (lead), formal analysis (lead), investigation (lead), methodology (lead), project administration (supporting), supervision (supporting), visualization (lead), writing – original draft (lead), writing – review and editing (equal). **Ernest E. Abano:** conceptualization (equal), data curation (supporting), formal analysis (supporting), investigation (supporting), methodology (supporting), project administration (supporting), supervision (lead), validation (lead), visualization (supporting), writing – review and editing (supporting). **Ernest Teye:** conceptualization (equal), data curation (supporting), methodology (supporting), project administration (supporting), supervision (supporting), validation (supporting), writing – review and editing (supporting). **Mary Lukeman:** investigation (supporting), methodology (supporting), project administration (supporting), resources (supporting), writing – review and editing (supporting). **Isaac Tukwarlba:** data curation (supporting), methodology (supporting), writing – review and editing (supporting). **Wilson Yeboah:** investigation (supporting), methodology (supporting), project administration (supporting), writing – review and editing (supporting). **David Nukafo:** investigation (supporting), methodology (supporting). **Kesse J. Agyei:** investigation (supporting), methodology (supporting).

## Ethics Statement

The authors have nothing to report.

## Conflicts of Interest

The authors declare no conflicts of interest.

## Data Availability

The data used to support the findings of this study are included within the article. However, any other information required is available from the corresponding author upon request.
